# Exploring natural therapy for chronic heart failure: experience in traditional Chinese medicine treatment before 2022

**DOI:** 10.3389/fmed.2025.1522163

**Published:** 2025-04-07

**Authors:** Li-Ping Pan, Lanxin Zhu, Bing-Xue Wang, Yi-Qi Li, Li Gao, Hui Hui Zhao

**Affiliations:** ^1^Institute of Ethnic Medicine and Pharmacy, Beijing University of Chinese Medicine, Beijing, China; ^2^School of Traditional Chinese Medicine, Beijing University of Chinese Medicine, Beijing, China; ^3^School of Traditional Chinese Medicine, Jinan University, Guangzhou, Guangdong, China

**Keywords:** CHF, symptom, traditional Chinese medicine, medical records, association rule, Apriori algorithm

## Abstract

**Background:**

Traditional Chinese medicine has great advantages in improving symptoms of CHF such as chest tightness, shortness of breath, and fatigue. In addition, some traditional Chinese medicines can be used as both medicine and food, which have good effects on the prevention and treatment of CHF patients at home.

**Method:**

A comprehensive search across China National Knowledge Infrastructure (CNKI), Wanfang, and Wei Pu (VIP) databases was conducted to retrieve pre-2022 literature related to CHF. After standardization, frequency analysis and Apriori algorithm were used to analyze these data.

**Result:**

Among 626 effective medical records, Fuling, Huangqi, and Danshen are the most commonly used herbs; The medication for chest tightness is closely related to Tinglizi; The medication for palpitations is closely related to Guizhi, Fuzi, Zhigancao, and Wuweizi; The medication of fatigue and poor appetite is closely related to Huangqi and Baizhu; The medication for lower limb edema is closely related to Fuling and Tinglizi; The medication for coughing is closely related to the use of Tinglizi, Wuweizi, Kuxingren, and Sangbaipi; Insomnia is closely related to Suanzaoren and Dazao.

**Conclusion:**

The components in traditional Chinese medicine that have anti heart failure effects and reliable evidence can be potential candidates for drug discovery, while dietary therapeutic herbs such as Fuling, Huangqi, Danshen, and Zhigancao can be developed as health products.

## 1 Introduction

CHF is a clinical syndrome caused by cardiac dysfunction, which is a chronic disease characterized by inadequate perfusion and congestion of tissues and organs throughout the body due to myocardial dysfunction, and cannot meet the metabolic needs of the body (1). The typical clinical manifestations are chest tightness, fluid retention, dyspnea, fatigue, etc. According to statistics, the global prevalence of heart failure among adults is 1 to 2%, and it increases with age (2–5). In the ESC guidelines, Sodium Glucose Gotransporter 2 Inhibitor (SGLT-2i) is recommended as the first-line medication for heart failure patients with reduced ejection fraction, along with the “golden triangle” drug, becoming the “new quadruple” drug for the treatment of HFrEF (6). The precursor and discovery of SGLT-2i class drugs were originally derived from the active ingredient of plant bark glycosides. As early as 1835, French chemists extracted a substance called bark glycosides from apple tree roots (7), indicating that natural products have great potential for development. Despite the continuous updates and developments in modern medical diagnosis and treatment technologies, patient mortality and readmission rates remain high (8, 9).

Natural products are a valuable source for the discovery of new drugs and health products. And promising natural medicines can be identified from Chinese herbal medicine, such as the well-known artemisinin, which was extracted from *Artemisia annua* based on inspiration from traditional Chinese medicine literature records. In China, the combination of traditional Chinese and Western medicine is a popular adjunctive treatment method. Both basic and clinical experimental studies have shown that the combination of traditional Chinese and Western medicine has achieved good results in the treatment of CHF (10). At present, studies have shown that the addition of traditional Chinese patent medicines and simple preparations “Qiliqiangxin capsule” on the basis of standard treatment can reduce the level of biomarker NT proBNP, improve heart function grading and alleviate symptoms (11). According to a systematic meta-analysis (12), the combination of traditional Chinese and Western medicine is superior to Western medicine alone in improving hemodynamic indicators in patients with CHF, and is safe and effective.

Modern clinical medical cases contain valuable records of traditional Chinese medicine’s experience in treating CHF. However, at present, there are either relatively few years of research and insufficient sample sizes in the use of traditional Chinese medicine for the treatment of CHF (13), or studies on a certain type of CHF (14), with insufficient coverage to explain the problem. There are few researchers on the symptoms of CHF and the corresponding medication for each symptom. Therefore, we have launched this data mining project to search for herbs that may improve different symptoms of CHF and provide a basis for selecting traditional Chinese medicine in clinical practice.

## 2 Materials and methods

**Figure 1:**
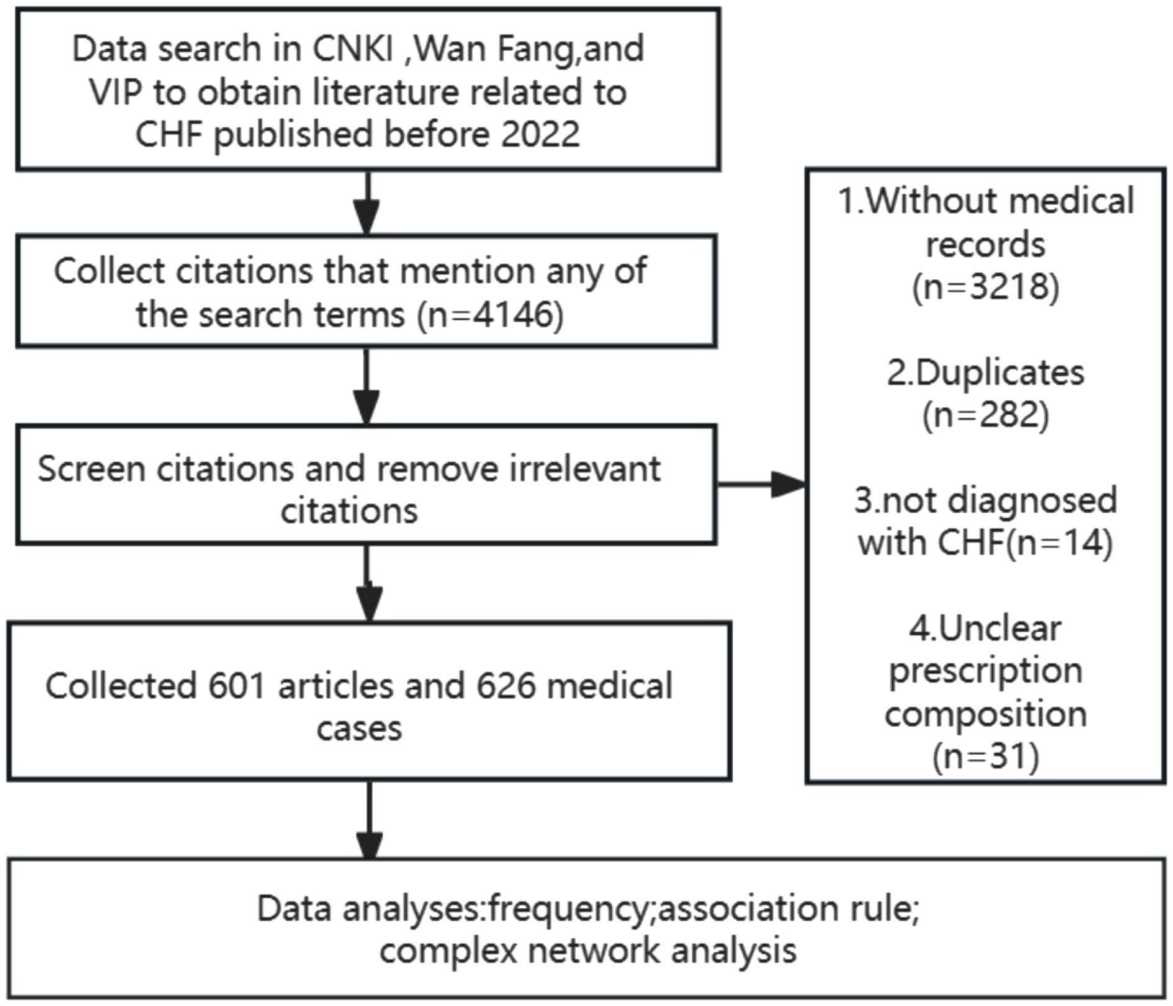
Flow chart of the research procedure.

Data mining is conducted in databases such as China National Knowledge Infrastructure (CNKI), Wanfang, and Wei Pu (VIP), which are the most influential academic databases in China and contain rich information on Traditional Chinese Medicine medical cases (Figure 1).

### 2.1 Search terms

The final search terms used in this study were determined by experts through discussion: the Chinese Pinyin of the search terms are “xin li shuan jie and jing yan,” “xin shuai and jing yan,” “xin li shuan jie and yi an,” “xin shuai and yi an,” “xin li shuan jie and yan an,” and “xin shuai and yan an.” Enter these terms into the search box to obtain relevant citations.

### 2.2 Eligible criteria

#### 2.2.1 Inclusion criteria

(1) Contains specific and effective traditional Chinese medicine cases.
(2) Diagnosed with CHF.
(3) Recorded changes in patient symptoms before and after treatment.
(4) Specific prescriptions are listed in the Chinese medicine.

#### 2.2.2 Exclusion criteria

(1) No specific medical records available.
(2) Duplicated citations.
(3) Subject not diagnosed with CHF.
(4) Literature with unknown prescription ingredients.

### 2.3 Citation screening

Two researchers independently conducted literature screening and data extraction based on inclusion and exclusion criteria, and resolved differences through discussion. The filtered and extracted data is input into Microsoft Excel 2019 to establish a database, including symptoms, formula composition, diagnosis, etc.

### 2.4 Data synthesis

The symptoms and medication of CHF in the citation were extracted and standardized. The standardization of Traditional Chinese Medicine names is based on the Pharmacopeia of the People’s Republic of China 2020. When citing a list of herbs without a formula name, they will be named after the formula containing the same ingredients. The standardization of symptoms was based on the WHO International Standard for Traditional Chinese Medicine Terminology.

### 2.5 Analytical methods

The Apriori algorithm is one of the most classic algorithms in association rule analysis. Simply enter the symptoms and traditional Chinese medicine according to the format, and you can see the calculation results from the association rule analysis option on the ancient and modern medical case cloud platform. In the Apriori algorithm, three standard metrics – support, confidence, and improvement – are used to measure the correlation between items. Support degree represents the probability of an itemset appearing in the total itemset; the higher the support, the higher the frequency of herbal appearance. Confidence reflects the probability of both the posterior and posterior terms appearing together in a dataset, while elevation represents the likelihood of an increase in the posterior term given a specific anterior term. We use the ancient and modern medical case cloud platform to perform statistical analysis, association rule analysis, and other data mining algorithms on the processed data, calculating the frequency of occurrence of traditional Chinese medicine and the degree of association between traditional Chinese medicine and symptoms. The combination of high-frequency herbs indicates that they have the potential to become a promising treatment method.

## 3 Results

A total of 4,146 articles were obtained, excluding 3,218 articles lacking complete medical records, 282 duplicate articles, 14 articles without clear diagnosis of CHF, 31 articles with unclear drug composition, and the remaining 601 articles that meet the requirements. Among them, a total of 626 qualified medical records were obtained.

### 3.1 Frequency analysis

#### 3.1.1 The frequency of the appearance of Chinese herbal medicine

Table 1 lists the top 10 herbs with the highest frequency of use, with Fuling being the most frequently used herb with a frequency of 458 times. Next are Huangqi, Danshen, Guizhi, Fuzi, Tinglizi, Zexie, Baizhu, Zhigancao, Maidong. It is worth noting that the top three Traditional Chinese Medicines have the functions of diuresis, tonifying qi, and promoting blood circulation, which coincides with the ideas of many experienced Chinese medicine practitioners in treating CHF (15–17).

**Table 1:** Most likely commonly used drugs for CHF.

Serial number	Herb name in Pinyin	Frequency (%)	Latin name
1	Fuling	480 (76.68%)	*Poria cocos*
2	Huangqi	338 (53.99%)	*Astragalus membranaceus*
3	Danshen	315 (50.32%)	*Salvia miltiorrhiza*
4	Guizhi	283 (45.21%)	*Ramulus Cinnamomi*
5	Fuzi	274 (43.77%)	*Aconitum carmichaeli*
6	Tinglizi	256 (40.89%)	*Descurainia sophia*
7	Zexie	246 (39.30%)	*Alisma orientalis*
8	Baizhu	243 (38.82%)	*Atractylodis Macrocephalae Rhizoma*
9	Zhigancao	187 (29.87%)	*Radix Glycyrrhizae Preparata*
10	Maidong	179 (28.59%)	*Ophiopogon japonicus*

#### 3.1.2 Frequency of symptom occurrence

Table 2 lists the frequency of common symptoms of CHF. The most common symptom is chest tightness, which occurs 396 times, followed by shortness of breath, palpitations, lower limb edema, fatigue, poor appetite, oliguria, cough, insomnia, inability to lie flat.

**Table 2:** Common symptom frequency of CHF.

Serial number	Symptoms of CHF	Frequency
1	Chest tightness	396
2	be short of breath	345
3	palpitate	336
4	Edema of Lower Extremities	252
5	feeble	182
6	poor appetite	146
7	oliguria	142
8	cough	139
9	insomnia	135
10	Difficulty lying flat	134

### 3.2 Analysis of association rules

**Figure 2:**
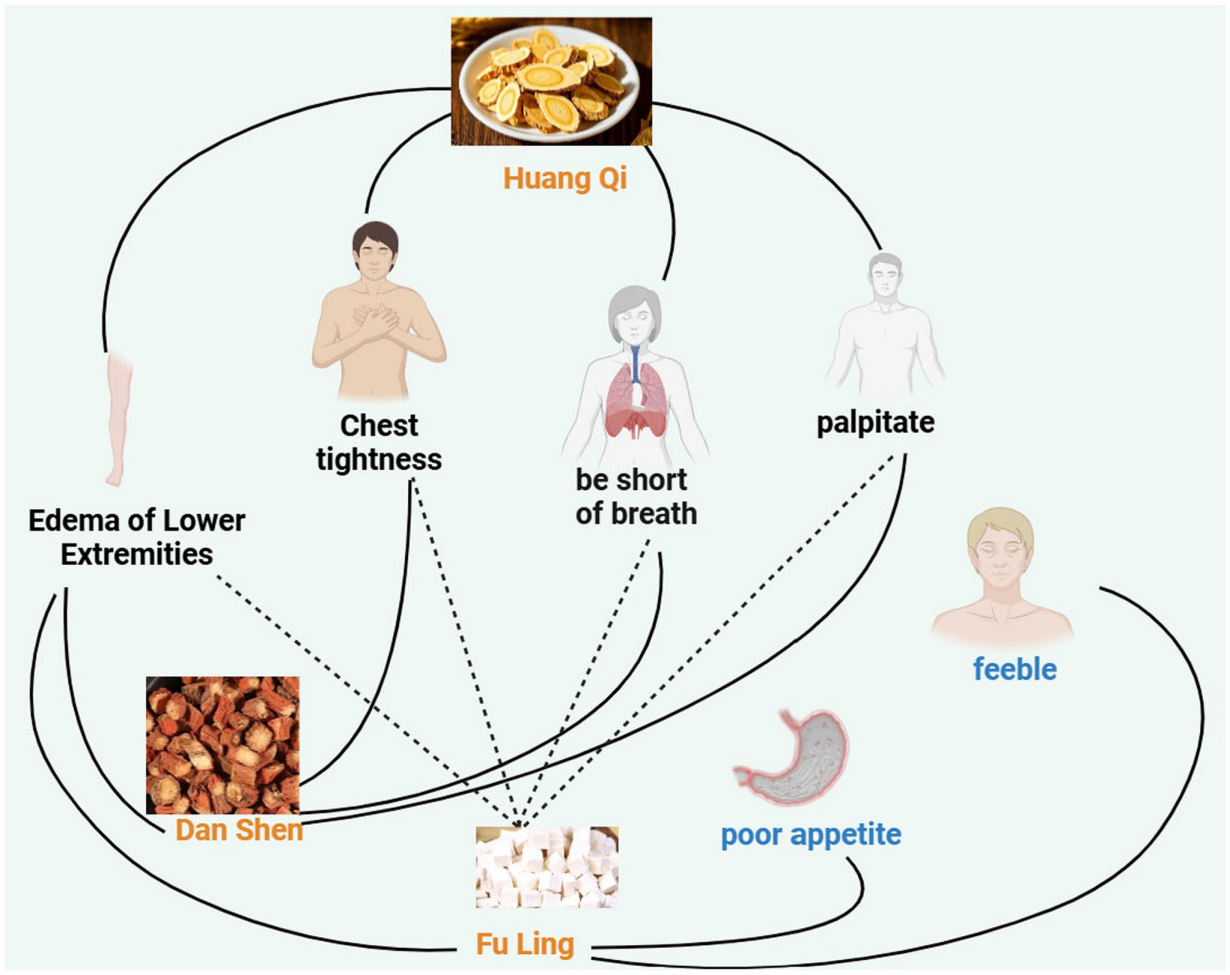
The correlation between traditional Chinese medicine and symptoms.

Symptoms are used as the front item set, while Traditional Chinese Medicine is used as the back item set. The support level is set to ≥20%, and the confidence level is set to ≥50%. As shown in Figure 2 and Table 3, symptoms such as chest tightness, palpitations, lower limb edema, and shortness of breath are closely related to Traditional Chinese Medicine such as Huangqi, Fuling, and Danshen. Symptoms such as fatigue and poor appetite are closely related to Fuling.

**Table 3:** Ranking of confidence levels related to traditional Chinese medicine and symptoms.

Serial number	Symptoms	Herb name	Support	Confidence	Lift
1	Chest tightness	Fuling	0.5	0.78	1.02
2	be short of breath	Fuling	0.43	0.78	1.02
3	palpitate	Fuling	0.42	0.79	1.03
4	Chest tightness	Huangqi	0.37	0.58	1.07
5	Chest tightness	Danshen	0.36	0.57	1.13
6	Edema of Lower Extremities	Fuling	0.32	0.78	1.02
7	be short of breath	Huangqi	0.31	0.56	1.04
8	palpitate	Huangqi	0.29	0.54	1.0
9	palpitate	Danshen	0.29	0.53	1.05
10	be short of breath	Danshen	0.28	0.51	1.01

We select 10 high-frequency symptoms of CHF and analyze the traditional Chinese medicine used. Set the support level to 0.2 and confidence level to 0.1, with the first item set being symptoms and the second item set being traditional Chinese medicine. Analyze using the association rules of the ancient and modern medical case cloud platform. Although the support and confidence levels have decreased, there will be an increase in potential traditional Chinese medicines for treating this symptom. This is also our innovation. The summary table of CHF symptoms and traditional Chinese medicine is as Table 4.

**Table 4:** Correspondence between symptoms and traditional Chinese medicine.

Symptoms	Chinese herbal medicine
Chest tightness	Fuling, Huangqi, Danshen, Tinglizi, Maidong, Guizhi, Zexie, Fuzi, Baizhu
Be short of breath	Fuling, Huangqi, Danshen, Guizhi, Fuzi, Tinglizi, Baizhu, Zexie
Palpitate	Fuling, Danshen, Huangqi, Guizhi, Fuzi, Baizhu, Zexie, Tinglizi, Maidong, Zhigancao, Wuweizi, Cheqianzi, Fuling, Dangshen, Chuanxiong, Danggui, Mudanpi
Edema of Lower Extremities	Fuling, Tinglizi, Danshen, Huangqi, Guizhi, Fuzi, Zexie, Baizhu, Fuling, Wuweizi, Zhigancao, Cheqianzi, Maidong, Dangshen
Feeble	Fuling, Huangqi, Danshen, Guizhi, Tinglizi, Baizhu, Fuzi, Zexie, Maidong, Zhigancao, Fuling, Dangshen, Wuweizi, Chuanxiong
Poor appetite	Fuling, Huangqi, Danshen, Baizhu, Zexie, Tinglizi, Guizhi, Fuzi, Zhigancao, Dangshen, Maidong, Wuweizi, Chuanxiong, Danggui
Cough	Fuling, Tinglizi, Guizhi, Fuzi, Danshen, Zexie, Baizhu, Fuling, Zhigancao, Wuweizi, Gancao, Kuxingren, Sangbaipi, Maidong
Oliguria	Fuling, Danshen, Huangqi, Zexie, Fuzi, Tinglizi, Baizhu, Guizhi
Insomnia	Fuling, Huangqi, Guizhi, Danshen, Tinglizi, Zexie, Baizhu, Zhigancao, Fuzi, Chuanxiong, Zhigancao, Wuweizi, Fuling, Danggui, Gancao, Dangshen, Cheqianzi, Taizishen, Yimucao, Suanzaoren, Honghua, Dazao
Difficulty lying flat	Fuling, Baizhu, Fuzi, Huangqi, Danshen, Guizhi, Zexie, Tinglizi

Compared with common data mining, our data mining has added high-frequency Chinese herbal medicines corresponding to symptoms. Most of the high-frequency Chinese herbal medicines corresponding to symptoms are Fuling, Huangqi, and Danshen. This may be related to the medication strategy of CHF, which mainly focuses on promoting water circulation, supplementing qi, and promoting blood circulation; The fourth highest frequency traditional Chinese medicine and subsequent traditional Chinese medicine have high specificity in the treatment of this symptom. For example, the fourth highest frequency medication for chest tightness is Tinglizi, which is consistent with the traditional Chinese medicine theory that Tinglizi has the function of reducing chest pressure and diuresis, thereby improving the symptoms of chest tightness.

Among the ten common symptoms of CHF, the potential medication for treating chest tightness is Tinglizi, without the use of Shaoyao, which is consistent with the conclusion of removing Shaoyao from chest tightness recorded in *Shanghan lun*——A classic book of traditional Chinese medicine. The potential drug for treating shortness of breath is Guizhi. According to traditional Chinese medicine theory, Guizhi has a “qi lowering” effect. The potential drugs for treating palpitations include Guizhi, Fuzi, Zhigancao, etc. There is a strong correlation between symptoms of lower limb edema and oliguria. In clinical practice, patients with lower limb edema can improve their symptoms of oliguria and lower limb edema after treatment with diuretics. In this study, the combination of traditional Chinese medicine such as Fuling, Tinglizi, Danshen, Huangqi, and Guizhi has potential diuretic effects. It is worth noting that the medication order for cough is different from other symptoms. The top three medications for other symptoms are Fuling, Huangqi, and Danshen. Huangqi is not included in the high-frequency medication order for cough, and Danshen ranks lower. The top ranked ones are Tinglizi, Guizhi, and Fuzi. In addition, the lower ranked ones such as Wuweizi, Kuxingren, and Sangbaipi are recognized as phlegm resolving, cough relieving, and asthma relieving drugs in traditional Chinese medicine. At the same time, it can also be seen that the lower ranked traditional Chinese medicine has a certain reference for improving symptoms. The potential drugs for treating insomnia include Guizhi, Tinglizi, Suanzaoren, Dazao, etc. The causes of insomnia are relatively complex, and medication is for reference only. Potential drugs for treating inability to lie flat include Baizhu, Fuzi, Huangqi, Danshen, Guizhi, Zexie, and Tinglizi.

### 3.3 Complex network analysis

**Figure 3:**
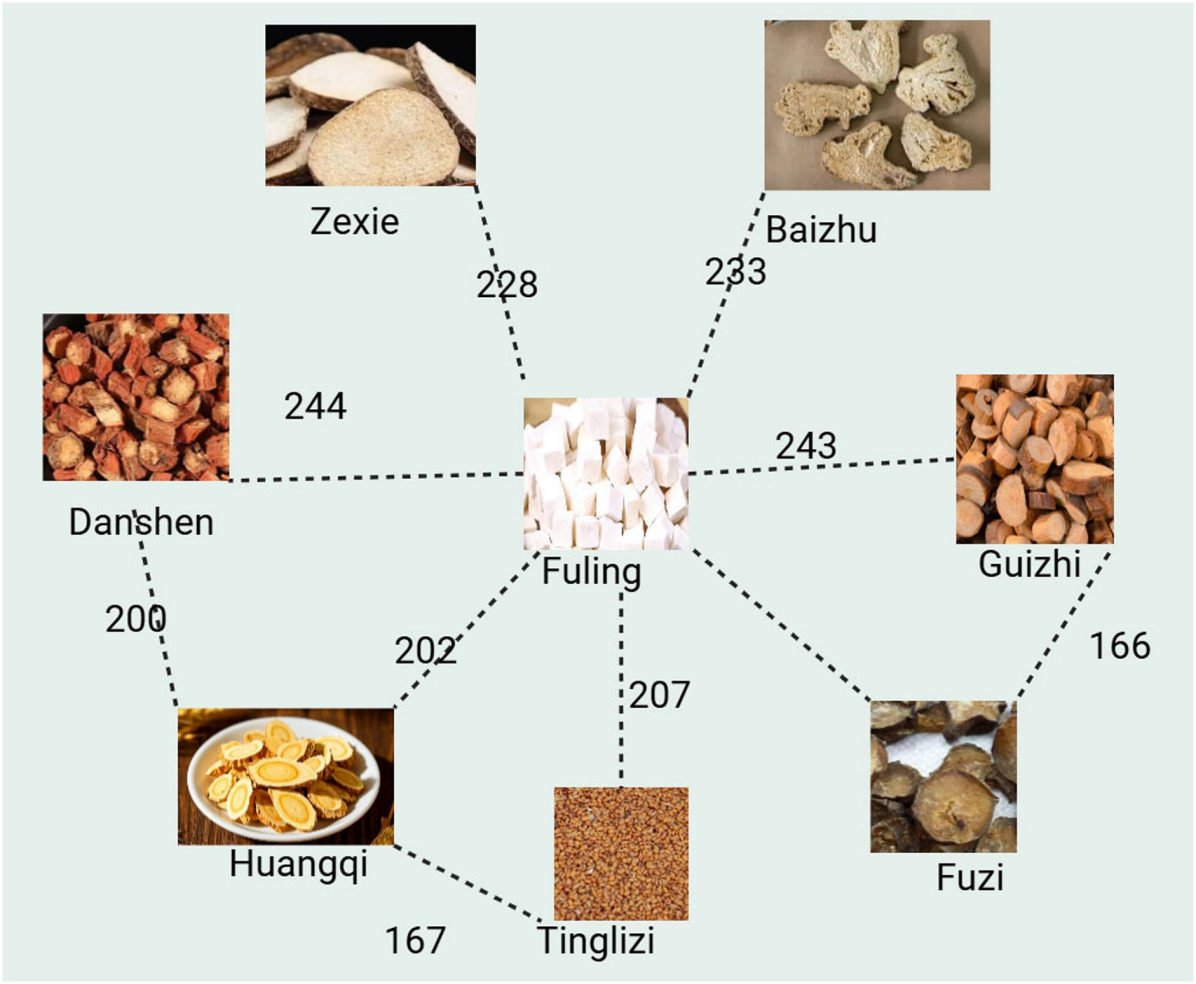
Traditional Chinese medicine – complex network analysis.

Select “Complex Network Analysis” from the ancient and modern medical case cloud platform, apply hierarchical network algorithm to analyze the relationship between traditional Chinese medicine and herbal medicine, set “Node Distance” to 15, Node Size to 50, Edge Weight to 15, Display Edge Count to 10, visualize the network, and output the network relationship diagram between traditional Chinese medicine and herbal medicine. Create a network diagram of traditional Chinese medicine as shown in Figure 3. Conclusion: The Fuling shown in Figure 3 is compatible with Huangqi, Danshen, Guizhi, Fuzi, Tinglizi, Baizhu, Zexie, and appears more than 200 times together. In addition, the compatibility between Danshen and Huangqi, Huangqi and Tinglizi, and Fuzi and Guizhi is also high.

## 4 Discussion

### 4.1 Summary of results

This study rigorously employs modern diagnostic criteria for CHF, ensuring the accuracy of medical record citations. Expert-derived search terms contribute to the reliability of the results. The findings reveal that Fuling, Huangqi, and Danshen emerge as the most frequently utilized herbs for CHF, aligning with existing research (14). Importantly, these versatile substances, serving both as drugs and foods, hold significant potential in the development of preventive health foods for CHF (18). In terms of the correspondence between the symptoms of heart failure and traditional Chinese medicine, the medication for chest tightness symptoms is closely related to Tinglizi; the medication for shortness of breath symptoms is closely related to Guizhi. The medication for symptoms of fatigue and poor appetite is closely related to Huangqi and Baizhu; the medication for lower limb edema symptoms is closely related to Fuling and Tinglizi; the medication for coughing is closely related to the use of Tinglizi, Wuweizi, Kuxingren, and Sangbaipi.

### 4.2 Action of herbs

Fuling, Huangqi, and Danshen, identified as high-frequency drugs for CHF treatment, exhibit commendable effects on heart failure through various pharmacological pathways. Fuling derived from saprophytic fungi in pine plants, it offers diuretic, sedative, and nourishing effects in traditional Chinese medicine. Triterpenoids and polysaccharides in Fuling extract and Fuling acid enhance heart function by inhibiting the renin-angiotensin-aldosterone system, regulating aquaporin expression, and inhibiting Na + -K-ATP activity (19–21). Huangqi renowned for improving myocardial energy metabolism, enhancing myocardial contractility, protecting blood vessels, and inducing diuresis (22–24).

Danshen is a well-known herbal medicine for treating cardiovascular diseases, but its quality evaluation and testing need to be strictly controlled (25). Studies have shown that the pharmacological effects of tanshinone mainly involve the expression of autophagy related proteins to improve cardiovascular disease (26).

Danshen rich in liposoluble active ingredients like tanshinone IIA, it enhances blood microcirculation, prevents thrombosis, strengthens the heart, and dilates blood vessels (27, 28). Tinglizi exhibits significant diuretic effects, with quercetin, kaempferol, and *β*-sitosterol among its main components. The AGE-RAGE signaling pathway, HIF-1 signaling pathway, and estrogen signaling pathway contribute to its comprehensive treatment of CHF (29, 30). Fuzi derived from the lateral root of Aconitum plants, it contains aconitine alkaloids with both pharmacological activity and toxicity. Water-soluble alkaloid extract (AWA) from Fuzi, containing aconitine and neoaconitine, improves CHF, but careful dosage control is essential due to its toxicity (31–34).

### 4.3 Limitations and future directions

While the data mining approach based on China National Knowledge Infrastructure (CNKI), Wanfang, and Wei Pu (VIP) databases provides substantial literature, the potential omission of individual studies remains a limitation. Additionally, the focus on published records may exclude effective yet unpublished data. Despite these limitations, the study offers valuable insights into potential effective herbs for CHF treatment.

### 4.4 Innovative contributions

The innovative contribution of this study lies in its meticulous application of modern diagnostic criteria, expert derived search terms, and comprehensive data mining to establish a subtle understanding of traditional Chinese medicine in the context of CHF. The correspondence between symptoms and traditional Chinese medicine, identification of high-frequency herbs, pharmacological effects, provide new insights into potential pathways for the treatment and prevention of health measures in CHF. The study also emphasizes the necessity of further exploring the convergence of medicinal and dietary benefits, laying the foundation for future research and development in this field.

Compared to previous articles, there are these breakthroughs:

*Innovative thinking*: Most common text mining articles explore medication frequency analysis, symptom association rule analysis with traditional Chinese medicine, high-frequency formula analysis, complex network analysis, and other aspects. In addition to the above analysis methods, this study also conducts frequency analysis on the traditional Chinese medicine used for high-frequency symptoms, thereby identifying the specificity and potential Chinese medicine corresponding to common symptoms of CHF. This approach is not found in traditional text mining.

*Method and result innovation*: This study screened common symptoms of CHF and conducted a correlation analysis between symptoms and drugs through the “Ancient and Modern Medical Case Cloud Platform.” The high-frequency drugs that appeared in a certain symptom were sorted to discover the relationship between specific symptoms and specific drugs. This result is highly similar to the traditional Chinese medicine’s understanding of drugs, such as Guizhi, which is a high-frequency drug for shortness of breath. Modern Chinese medicine textbooks do not mention that Guizhi can treat shortness of breath, but in the ancient Chinese medicine book “Shennong Bencao Jing,” it is mentioned that Guizhi can treat “cough reflux and upper qi”; In addition, there is no mention of Shaoyao in the medication statistics for chest tightness, which is consistent with the traditional Chinese medicine’s approach to removing Shaoyao from chest tightness, indirectly confirming the viewpoint of traditional Chinese medicine; Huangqi is not included in the medication statistics for cough. According to the high-frequency medication for heart failure, Fuling, Huangqi, and Danshen are the top three drugs. However, when symptoms of cough occur, Huangqi does not appear in the high-frequency medication. This also raises a new hypothesis: patients with CHF should use Huangqi with caution when coughing. Traditional Chinese medicine believes that using Huangqi during coughing can lead to the retention of evil energy in the body, which can easily worsen coughing. On the contrary, Tinglizi has replaced the order of Huangqi and appeared in the high-frequency medication for coughing. There are many similar new discoveries in this study.

## 5 Conclusion

Fuling, Huangqi, and Danshen are the most commonly used drug combinations. In terms of the corresponding symptoms of heart failure and traditional Chinese medicine, the medication for chest tightness symptoms is closely related to the use of Tinglizi; The medication for shortness of breath symptoms is closely related to Guizhi; The medication for symptoms of fatigue and poor appetite is closely related to Huangqi and Baizhu; The medication for lower limb edema symptoms is closely related to Fuling and Tinglizi; The medication for coughing is closely related to the use of Tinglizi, Wuweizi, Kuxingren, and Sangbaipi. In addition, traditional Chinese medicines such as Fuling, Zhigancao, Huangqi, and Dazao, which have the same medicinal and dietary origin, have the potential for further evaluation as self-management nutritional supplements for patients with CHF.

## Data Availability

The original contributions presented in the study are included in the article/Supplementary material, further inquiries can be directed to the corresponding author.
