# Supplementary material for: Exploring natural therapy for chronic heart failure: experience in traditional Chinese medicine treatment before 2022
Source: Front Med (Lausanne). 2025 Apr 7;12:1522163. doi: 10.3389/fmed.2025.1522163 (PMC12013336; doi:10.3389/fmed.2025.1522163)
Supplement: Supplementary file 1 [file Data_Sheet_1.pdf]

Reference Table for Pinyin and Latin Names of Chinese Herbal Medicine

Serial Number	Pinyin	Latin name
1	Fuling	*Poria cocos*
2	Huangqi	*Astragalus membranaceus*
3	Danshen	*Salvia miltiorrhiza*
4	Tinglizi	*Descurainia sophia*
5	Guizhi	*Ramulus Cinnamomi*
6	Fuzi	*Aconitum carmichaeli*
7	Zhigancao	*Radix Glycyrrhizae Preparata*
8	Wuweizi	*Schisandra chinensis*
9	Kuxingren	*Prunus armeniaca var. ansu*
10	Sangbaipi	*Mori Cortex*
11	Suanzaoren	*Ziziphi Spinosae Semen*
12	Dazao	*Ziziphi Jujubae Fructus*
13	Maidong	*Ophiopogon japonicus*
14	Zexie	*Alisma orientalis*
15	Cheqianzi	*Semen Plantaginis*
16	Dangshen	*Codonopsis pilosula*
17	Danggui	*Angelica sinensis*
18	Mudanpi	*Paeonia suffruticosa*
19	Gancao	*Glycyrrhiza uralensis*
20	Chuanxiong	*Ligusticum chuanxiong*
21	Taizishen	*Pseudostellaria heterophylla*
22	Yimucao	*Leonurus japonicus*
23	Honghua	*Carthami Flos*
24	Shaoyao	*Paeoniae Radix Alba*
25	Baizhu	*Atractylodis Macrocephalae Rhizoma*
